# Immune Characteristics and Immunotherapy of HIV-Associated Lymphoma

**DOI:** 10.3390/cimb46090596

**Published:** 2024-09-10

**Authors:** Yi Liu, Xiaoqing Xie, Jun Li, Qing Xiao, Sanxiu He, Huihui Fu, Xiaomei Zhang, Yao Liu

**Affiliations:** 1 School of Medicine, Chongqing University, Chongqing 400030, China; 2 Department of Hematology-Oncology, Chongqing University Cancer Hospital, Chongqing 400030, China

**Keywords:** HIV, HIV-associated lymphoma, immune system, tumor micro-environment, immune therapy

## Abstract

In the era of antiretroviral therapy (ART), mortality among people living with the human immunodeficiency virus (HIV) has significantly decreased, yet the population of people living with HIV remains substantial. Among people living with HIV (PLWH), HIV-associated lymphoma (HAL) has surpassed Kaposi’s sarcoma to become the most common tumor in this population in developed countries. However, there remains a dearth of comprehensive and systematic understanding regarding HIV-associated lymphomas. This review aims to shed light on the changes in the immune system among PLWH and the characteristics of the immune microenvironment in HIV-associated lymphoma, with a specific focus on the immune system’s role in these individuals. Additionally, it seeks to explore recent advancements in immunotherapy for the treatment of HIV-associated lymphoma, intending to enhance strategies for immunotherapy in this specific population.

## 1. Introduction

With the widespread use of ART, the incidence of HIV-associated opportunistic infections has significantly decreased, while malignant tumors have emerged as the leading cause of death in people living with HIV (PLWH) within many developed countries.

Over the last 20 years, the incidence of certain HIV-associated lymphomas has drastically decreased. Diffuse large B-cell lymphoma (DLBCL) and primary central nervous system lymphoma (PCNSL) have experienced a drastic decrease, whereas Burkitt lymphoma (BL) has remained stable, and Hodgkin lymphoma (HL) has increased [1]. The 5th edition of the World Health Organization classification of lympho-hemopoietic neoplasms (WHO-HAEM5) included a specific classification of lymphomas caused by HIV infection [2]. A comprehensive understanding of the immunological characteristics of HIV-associated lymphomas (HALs) will not only deepen our understanding of the intrinsic relationship between HIV and HALs but also provide valuable insights for the development of immunotherapy strategies.

## 2. The Pathogenesis of HIV-Associated Lymphoma

HAL is a malignancy influenced by HIV via direct and indirect mechanisms. HIV proteins like trans-activating regulatory protein (Tat) and matrix protein p17 (p17) can directly drive malignant transformation by affecting oncogene expression and genomic stability. Tat impacts B cells, while p17 accumulates in lymphoma tissues, promoting development. Indirectly, HIV’s immunosuppressive effects disrupt immune function and cytokine secretion, aiding lymphoma progression. HIV-induced immune dysfunction impairs natural killer cells, T cells, and macrophages, and co-infection with viruses like Epstein–Barr (EBV) heightens risk. Understanding these mechanisms underscores the need for targeted HAL management strategies.

### 2.1. Direct Carcinogenesis

HIV cannot directly infect B cells, but HIV viral proteins secreted by HIV can enter bystander cells, including B cells, and drive their malignant transformation [3]. HIV proteins such as negative factor (Nef), Tat, and p17 can be detected in lymphomas and lymph nodes, and the detection rate of HIV proteins is higher in lymphomas and lymph nodes of HAL patients [4]. However, the mechanism by which HIV proteins enter B cells remains unclear and requires further investigation. In HIV transgenic mouse lymphoma models, spontaneous B-cell lymphomas can be induced by the expression of HIV p17 alone, without other HIV proteins [5]. Persistent accumulation of the HIV-1 p17 protein has also been observed in lymphomas and lymph node tissues of people living with HIV [4]. Studies have shown that HIV relies on phosphatidylinositol-4,5-bisphosphate (PI-4,5-P2) to secrete p17 variants (vp17s) [6], binding to protease-activated receptor 1 (PAR1) on the B-cell membrane, activating the PAR1/EGFR/PI3K/Akt pathway, and promoting HAL [7]. Tat can bind to activator protein 1 (AP-1) and JunB and activate the c-MYC promoter, upregulating oncogene expression in B cells [8]. Additionally, B cells exposed to Tat also exhibit increased frequencies of chromosomal aberrations, which promote lymphoma formation [9]. Furthermore, the ability of Tat to penetrate B cells spontaneously is sufficient to promote MYC-IGH oncogenic rearrangements during error-prone repair, which is a plausible reason for increased HAL incidence [10]. Tat also activates the Akt/mTORC1 signaling pathway, downregulates the activation-induced cytidine deaminase (AICDA) transcriptional repressors c-Myb and E2F8, induces the aberrant activation of AICDA, and increases B-cell genomic instability and malignant proliferation [11,12]. Similarly, HIV Nef leads to significant increases in activation-induced cytidine deaminase (AID) and c-MYC, causing genomic instability and enhancing the risk of B-cell development into BL [13].

**Figure 1:**
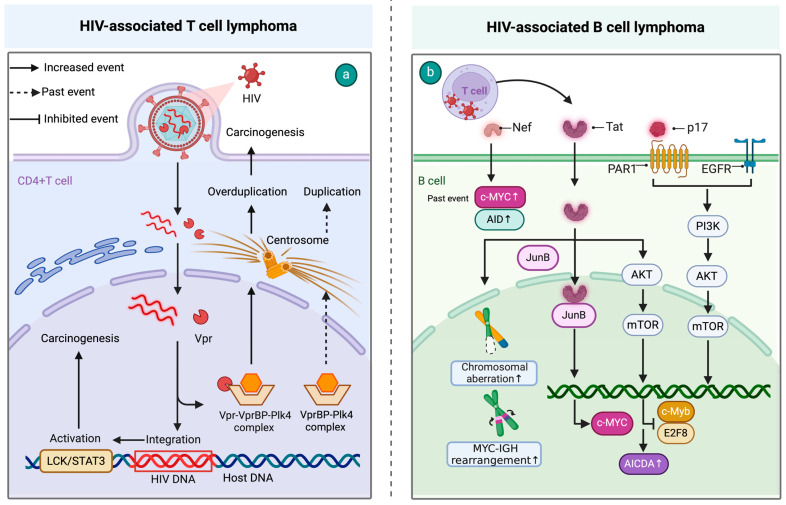
Mechanisms of HIV-induced lymphomagenesis. (**a**) Chronic HIV promotes T-cell malignancy via the Vpr-VprBP-Plk4 complex which induces centrosome amplification and aneuploidy. HIV-1 provirus integration into STAT3 and LCK disrupts normal signaling, further advancing T-cell lymphoma development. (**b**) HIV proteins such as p17 and Tat drive B-cell lymphomas by activating oncogenic pathways and disrupting genomic stability. p17 binds to PAR1 on B cells, triggering the PAR1/EGFR/PI3K/Akt pathway. Tat enhances c-MYC expression, causing chromosomal aberrations and MYC-IGH rearrangements, while also activating the Akt/mTORC1 pathway to increase genomic instability. HIV Nef further contributes by elevating AID and c-MYC, promoting Burkitt lymphoma risk. Created with BioRender.com.

T-cell HALs are less common than B-cell HALs, possibly because T-cell malignant transformation requires the accumulation of more mutations [14]. Recent studies have shown that chronic HIV infection can promote the process of malignant transformation in T cells. HIV primarily infects CD4^+^ T cells and induces the Vpr-VprBP-Plk4 complex, linking HIV-1 infection with T-cell tumorigenesis through centrosome amplification and aneuploidy [15]. Additionally, the integration of HIV-1 provirus into the first intron of signaling sensors and transcription activators (STAT3) and lymphocyte-specific protein tyrosine kinase (LCK) also plays a crucial role in the development of T-cell lymphomas [16]. In conclusion, HIV-induced mechanisms drive both B- and T-cell malignancies through various pathways, including protein interactions and genomic instability (see Figure 1).

### 2.2. Indirect Carcinogenesis

HIV infection directly damages the immune system, leading to immunosuppression and abnormal cytokine secretion, which are significant factors in the development and progression of HAL. CD4^+^ T cells and macrophages are critical target cells for HIV [17]. HIV-infected macrophages have a crucial impact on HIV latency and disease progression by causing chronic inflammation [18,19]. Bone marrow stromal antigen 2 (BST2), synthesized by macrophages, limits reverse transcription and impedes viral release [20,21]. However, HIV viral protein U (Vpu) targets BST2 for degradation, facilitating persistent HIV infection and chronic inflammation [22]. Additionally, HIV-infected macrophages exhibit high levels of miRNA-99, which inhibits the degradation of HIV, leading to macrophage pyroptosis and triggering the release of TNF-α, IL-6, and IL-1β, thereby causing chronic inflammatory activation [23,24]. Indoleamine 2,3-dioxygenase (IDO) is primarily sourced from antigen-presenting cells like macrophages and dendritic cells [25]. HIV Tat and Nef increase IDO expression [26], leading to Th17 cell depletion, Treg expansion [27], and reduced cytotoxic T-cell proliferation, which facilitates immune evasion [28]. Furthermore, miRNA-146a is also upregulated in HIV-infected macrophages, where it negatively regulates innate immune responses and downregulates CC chemokine ligand 5 (CCL5) [24]. HIV infection also suppresses the activation of CD56bright NK cells, resulting in decreased cytotoxicity and immune escape [29]. In HIV-infected individuals, the expression of nitrogen-centered radicals (NCRs) and NK receptor group 2 member D (NKG2D) is reduced post-transcriptionally, impairing NK cell recognition of pre-cancerous tissues [30,31].

CD4^+^ T cells infected with HIV show reductions in both quantity and function [32], while CD8^+^ T cells exhibit overexpression of the inhibitory receptors programmed cell death-1 (PD-1) and suppressor of cytokine signal 7 (SOCS-7), which impairs their killing function [33,34,35]. HIV Tat affects B cells by inhibiting NF-κB pathway activity, reducing MHC class II gene transcription, which hinders immune surveillance [36]. Furthermore, regardless of ART treatment, B-cell activating factor (BAFF) remains elevated in the peripheral blood of HIV-infected individuals compared to healthy controls, leading to significantly reduced expression of NR4A1, NR4A3, and CD83 in marginal zone precursor (MZp) B cells and decreased Breg function, thereby increasing the risk of marginal zone B lymphoma [37,38]. 

**Figure 2:**
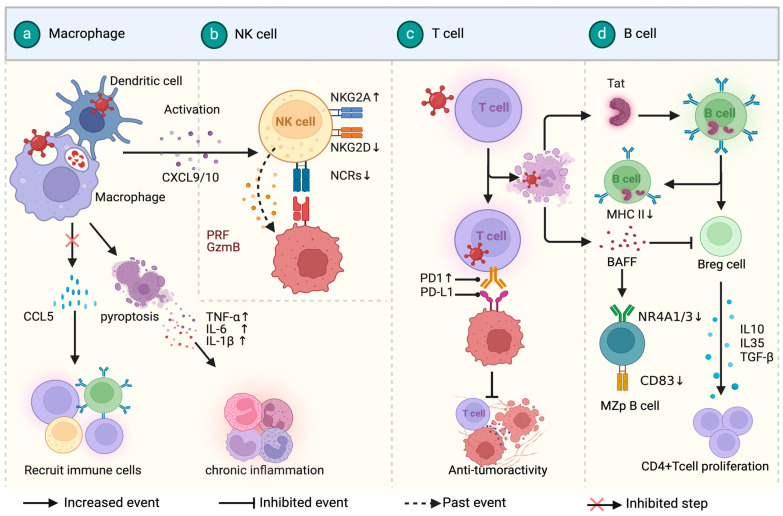
Mechanisms of HIV-induced lymphomagenesis. (**a**) HIV infection induces chronic inflammation and immune dysfunction, leading to lymphoma. HIV-infected macrophages impair BST2, triggering pyroptosis and elevated levels of miRNA-99 and miRNA-146a, disrupting immune responses. (**b**) HIV suppresses NK cell activity by downregulating activation receptors like NKG2D and upregulating inhibitory receptors such as NKG2A. (**c**) CD4^+^ T-cell depletion and CD8^+^ T-cell dysfunction, marked by PD-1 overexpression, contribute to reduced immune surveillance. (**d**) HIV also impairs B-cell function through Tat-induced NF-κB pathway inhibition and persistent BAFF elevation. Created with BioRender.com.

In addition to immune abnormalities, co-infection with other oncogenic viruses is also a potential mechanism for HALs. Epstein–Barr virus (EBV) is a major oncogenic virus driving lymphoma development [39]. HIV infection leads to T-cell depletion, facilitating EBV proliferation in infected B cells and resulting in the abnormal activation and malignant proliferation of B cells [40]. Moreover, in EBV co-infection conditions, B cells in humanized mice can also be infected with HIV-1, suggesting potential for B-cell HIV infection, though the exact mechanism remains unclear and lacks validation in people living with HIV [41]. Co-infection with HIV-1 and HTLV-1 results in a significantly higher clonal expansion of infected cells compared to infections with HIV or HTLV-1 alone, and this increased clonal expansion is one of the potential mechanisms for the elevated risk of developing Adult T-Cell Leukemia–Lymphoma (ATLL) [42]. HIV infection drives the development and progression of HALs by causing chronic inflammation, impairing immune cell function, and facilitating co-infection with oncogenic viruses like EBV and HTLV-1, ultimately leading to increased lymphoma risk and progression (see Figure 2).

## 3. Immune Microenvironment in HIV-Associated Lymphoma

Due to HIV infection-induced immune response damage, PLWH have a significantly higher risk of developing lymphomas compared to the general population. HALs exhibit distinct clinical presentations, histopathological characteristics, and prognosis. They are often associated with poorer prognosis, the involvement of extranodal sites or the central nervous system, and oncogenic infections.

The tumor immune microenvironment (TIME) in HALs is a dynamic interplay among tumor cells, immune cells, and the surrounding extracellular matrix [43]. It comprises tumor-associated macrophages (TAMs), NKs, T cells, B cells, oncogenic viruses, and cytokines, all contributing to the disease’s development. Recent evidence suggests that immune cells initially have an anti-tumor role during early tumor invasion but gradually transition to a pro-tumor phenotype, promoting immunosuppression, tumor immune escape, and distant metastasis as the tumor progresses [44]. Understanding the mechanisms involved in the TIME provides valuable insights into cancer biology and potential therapeutic strategies for HALs.

### 3.1. HIV-Associated Diffuse Large B-Cell Lymphoma

HIV-associated diffuse large B-cell lymphoma (DLBCL) is the most common form of HIV-associated lymphoma [45]. It often occurs in individuals with sustained viremia and profound immunosuppression, with a median CD4^+^ T-cell count of <200/μL at the time of diagnosis [46,47,48]. Its tumor microenvironment shows significant heterogeneity between the ABC (Activated B-cell-like) subtype and GCB (Germinal Center B-cell-like) subtype based on cell-of-origin (COO) classification. The ABC subtype is characterized by a gene expression profile resembling activated B cells and is often associated with poorer outcomes and increased EBV positivity [49]. Similarly, ABC subtype HIV-associated DLBCL is often EBV-positive, while the GCB subtype is typically EBV-negative [48,50,51]. HIV-associated DLBCL without EBV infection exhibits significantly higher copy number alterations compared to EBV-positive cases [52]. EBV-positive HIV-associated DLBCL occurs with lower CD4^+^ T-cell counts and is characterized by a higher frequency of recurrent STAT3 mutations [48]. The tissues of ABC-subtype HIV-associated DLBCL are more prone to extensive necrosis compared to the GCB subtype [53,54]. Both ABC- and GCB-like HIV-associated DLBCL exhibit a higher vascular density compared to DLBCL in the general population [55]. 

In HIV-associated DLBCL cases, the frequencies of MYC and BCL6 rearrangements (14.9% and 27.7%, respectively) are like those described in HIV-negative patients, but BCL2 rearrangements are less common (4.3%) [56]. Additionally, a specific study focusing on GCB-type HIV-associated DLBCL found increased expression of genes associated with cell cycle progression, DNA replication, and damage repair, while the expression of cell cycle inhibition and apoptosis-related genes was decreased [57]. These findings support the clinical observation that GCB-type HIV-associated DLBCL has higher proliferative potential and enhanced genomic stability. 

### 3.2. HIV-Associated Burkitt Lymphoma

Burkitt lymphoma (BL) is a rare and highly aggressive form of non-Hodgkin lymphoma, accounting for only 1–2% of all non-Hodgkin lymphoma cases. However, in PLWH, its prevalence increases significantly, constituting 25–40% of HALs. Remarkably, even with the advent of antiretroviral therapy (ART), the incidence of HIV-associated BL has not seen a decline. Patients diagnosed with HIV-associated BL tend to present with distinct clinical features compared to BL patients without HIV infection [58]. B symptoms in lymphoma refer to unexplained fevers, night sweats, and weight loss, which may indicate a more aggressive disease [59]. HAL patients are more likely to exhibit extranidal involvement, B symptoms, and a frailty status and elevated lactate dehydrogenase [60]. The tumor microenvironment in HIV-associated BL is marked by elevated CD4^+^ T-cell counts, a higher prevalence of EBV infection, universal MYC gene rearrangement, and the presence of HIV Tat protein, contributing to a more aggressive phenotype.

HIV-associated BL occurs in patients with higher CD4^+^ T-cell numbers than other HALs. It often arises in patients with CD4^+^ cell >200 cells/mm^3^, whereas HIV-associated PCNSL and PEL are more likely to be seen with CD4^+^ cell < 50 cells/mm^3^ [58]. The prevalence of EBV infection in HIV-positive BL cases was as high as 60%, while in HIV-negative cases, the EBV prevalence only reached 20% [61]. In line with endemic and sporadic BL, the rearrangement of the MYC gene on chromosome 8 is a nearly universal occurrence in HIV-associated Burkitt lymphoma [62]. The HIV Tat protein was found to be present in HIV-associated BL. The HIV Tat protein enhances the activity of the c-MYC gene promoter by binding to AP-1, thereby contributing to a more aggressive phenotype in HIV-associated Burkitt lymphoma [8]. Circulating soluble Tat protein in the serum of HIV-infected individuals can be internalized by non-host cells, which may explain the presence of Tat in B cells [63,64]. Additionally, in in vitro experiments, a significant downregulation of hsa-miR-200c-3p was observed in HIV-associated BL, leading to increased expression of Zinc-finger E-box-binding homeobox 1 (ZEB1) and ZEB2, conferring a stronger invasive capacity to HIV-associated BL. While the finding of this study has not been validated in patient tissue samples [65], it suggests that HIV may play a role in promoting the aggressiveness of BL through these mechanisms [13].

### 3.3. HIV-Associated Hodgkin Lymphoma

Although Hodgkin lymphoma (HL) in PLWH is not classified as an HIV-associated lymphoma in the latest version of the National Comprehensive Cancer Network (NCCN) guidelines [66], given its significantly increased incidence in PLWH, this article also provides a summary of the microenvironment of HIV-associated HL. The probability of developing HL in PLWH is 5–15 times higher than general population [67], and the use of ART increases this risk to about 20–30 times that of the general population [68]. Despite viral load control and CD4 recovery in HIV patients on ART, the risk of HL remains nine times higher than in the general population [69], suggesting that immune reconstitution after ART is associated with the development of HIV-related HL [70].

Research has revealed that Hodgkin lymphoma in PLWH exhibits a distinctive immunological microenvironment landscape. Firstly, what predominates in HIV-associated HL is an abnormal proportion of tumor cells known as Reed–Sternberg (R-S) cells [71]. When diagnosing HIV-associated HL, the median CD4^+^ cell count is relatively high, with a range of approximately 275–306 cell/μL [72]. The number of functional NK cells and mature NK cells in HIV-associated HL tissues is also lower compared to HIV-negative HL cases [73]. Some researchers have observed that macrophages in HIV-associated HL tend to express CD163^+^ and can surround R-S cells [74]. But their biological effects are not yet certain, and it is speculated that this might be related to frequent treatment failures following chemotherapy. Secondly, most HIV-associated HL cases express LMP1 and display the post germinal center B phenotype cells [72]. And approximately 80% to 100% of HIV-associated HL tissues exhibit EBV infection [75], while EBV infection is found in only 20–40% of Hodgkin lymphoma cases in the general population [76]. In nearly all cases of HIV-associated HL, RS cells carry the EBV genome and strongly express the latent membrane protein-1 (LMP-1) viral oncogenic protein [77]. Therefore, it is postulated that a cooperative interaction between EBV and HIV promotes the malignant proliferation of B cells in HIV-associated HL. Research indicates that HIV product p17 upregulates the expression of the EBV latency-specific product LMP-1 and that its accumulation in tumor tissues targets CXC chemokine receptor 2 (CXCR2) to enhance B-cell clonality [78]. Furthermore, chronic HIV infection induces the abnormal production of IL-6 and IL-10, leading to the uncontrolled clonal proliferation of B lymphocytes [79].

### 3.4. Other Rare HIV-Associated Lymphomas

Primary central nervous system lymphoma (PCNSL) is a highly invasive and rare tumor. The incidence of PCNSL is significantly increased in HIV-infected patients, accounting for 12–15% of HIV-related lymphomas. The majority of PCNSL cases (90–95%) are DLBCL, with a minority being Burkitt or T-cell lymphomas. EBV infection occurs in 80–100% of PCNSL. There is a causal relationship between Human Herpesvirus 8 and primary effusion lymphoma, and infection is a diagnostic requirement. The incidence of HIV-associated PBL accounts for approximately 2% of all HIV-associated lymphomas. Several case reports and case series have been published. The association between Human Herpesvirus 8 infection and plasmablastic lymphoma is not as clear. However, approximately 75% of HIV-associated PBL cases are EBV-positive. Additionally, about 50% of PBL cases exhibit MYC gene rearrangements or MYC gains and are CD20-negative. Currently, there are only a limited number of case reports on these rare HIV lymphomas, and further research is needed to thoroughly investigate their clinical and microenvironmental characteristics.

## 4. Immunotherapy of HIV-Associated Lymphomas

HAL presents distinct clinical characteristics and progression compared to common lymphomas. It is often diagnosed at an advanced stage, typically stage ≥III, and is more likely to involve extra nodal sites and present systemic symptoms [80]. These unique features require a tailored treatment approach. The most common treatment for HAL involves a combination of chemotherapy and ART. ART not only targets HIV but also enhances the tolerance to chemotherapy, allowing for standard-dose or high-dose chemotherapy regimens to be used [81]. According to the 5th version of the NCCN guidelines, the first-line therapy for HAL is summarized in Table 1 [66]. 

Although the concurrent administration of ART and chemotherapy is considered safe and practical, there is currently no standard treatment regimen for recurrent/refractory HAL. Given its close association with the pathogenesis and the immune system, researchers have made more attempts in immunotherapy. Immunotherapies for lymphoma include antibody therapy, chimeric antigen receptor T-cell (CAR-T) therapy, and immune checkpoint inhibitors [82]. These approaches aim to harness the immune system to recognize and eliminate cancer cells. Antibody therapy involves the use of monoclonal antibodies that specifically target antigens on lymphoma cells, leading to their destruction. CAR-T therapy involves genetically modifying a patient’s T cells to express chimeric antigen receptors that recognize and attack lymphoma cells. Immune checkpoint inhibitors block inhibitory pathways in the immune system, allowing T cells to mount a more robust anti-tumor response. These immunotherapeutic approaches hold promise in improving outcomes for HAL patients, particularly those who have relapsed or are refractory to standard treatments.

### 4.1. Monoclonal Antibody Therapies

Antibody immunotherapy has emerged as a promising approach in the treatment of HAL, particularly targeting the CD20 antigen expressed on B cells. Rituximab, an anti-CD20 monoclonal antibody, has shown promise in HAL patients. Since the majority of HIV-associated DLBCLs express CD20, the use of rituximab in combination with CHOP or EPOCH regimens has demonstrated a significant improvement in patient survival benefits, with a 5-year progression-free survival (PFS) rate reaching up to 87.8% and a 50% reduction in the risk of death [83]. It is noteworthy that, although HIV-associated PEL is often CD20-negative, NCCN guidelines still recommend the use of rituximab. This recommendation is due to the fact that most PEL patients also suffer from multicentric Castleman’s disease (MCD), and rituximab is the standard treatment for MCD [84,85]. Additionally, rituximab can target and eliminate B cells infected with KSHV, reducing the release of inflammatory factors that drive the natural progression of PEL [84]. Therefore, rituximab is recommended as a first-line treatment for HIV-associated PEL, but caution is needed for the potential development of Kaposi’s sarcoma during rituximab therapy [83].

Brentuximab vedotin (BV), an antibody–drug conjugate (ADC) composed of the anti-CD30 monoclonal antibody cAC10, has been reported to have a dual effect on HIV-related PEL, effectively controlling lymphoma and HIV infection [84]. In a reported case, an HIV-related PEL patient with a CD4 count of 29 cells/µL and an HIV viral load of 327,367 copies/mL achieved complete remission (CR) after six courses of BV combined with antiretroviral therapy. After 17 courses of BV combined with antiretroviral therapy, the CD4 count increased to 448 cells/µL, and the HIV viral load dropped below the detection limit. Despite the occurrence of grade 2 sensory peripheral neuropathy during treatment, reducing the dose of BV reversed this adverse reaction [84]. The mechanism of BV’s targeted clearance of HIV is likely based on the characteristic overexpression of CD30 in CD4^+^ T cells with latent HIV infection in a suppressive antiretroviral (AR) environment. In vitro experiments have demonstrated that Brentuximab vedotin significantly reduces the total amount of HIV-1 DNA in peripheral blood mononuclear cells from an infected, ART-suppressed environment, supporting the potential positive role of BV in clearing latent HIV infection [85].

CD38 monoclonal antibody daratumumab is a fully human IgG1 monoclonal antibody specifically targeting the CD38 molecule expressed on the surface of myeloma cells, exerting anti-myeloma activity through immune-mediated mechanisms, the direct induction of cell apoptosis, and immunomodulation [86]. Given the surface expression of CD38 on PBL, researchers have attempted to apply daratumumab to PBL. An HIV-related PBL patient achieved CR after receiving six cycles of daratumumab combined with EPOCH and maintained this response for 17 months, suggesting the potential efficacy of daratumumab in combination with the EPOCH regimen for HIV-related PBL [87]. However, it is essential to recognize that the research is still in its early stages, and the number of cases is limited. Future studies need to further validate these findings, including larger-scale clinical trials, to determine the exact efficacy and safety of these drugs in patients with HIV-related PBL.

### 4.2. Immune Checkpoint Inhibitors

Immune checkpoint inhibitors, such as PD-1/PD-L1 inhibitors and CTLA-4 inhibitors, have demonstrated encouraging potential in addressing resistant solid tumors by releasing immune restraints within the tumor microenvironment and reactivating T cell-mediated immune responses. These inhibitors have also been found to reverse the incubation period of HIV and enhance HIV-specific immunity. Recent research has demonstrated the safety and effectiveness of immune checkpoint inhibitors in the treatment of relapsed or refractory HAL (Table 2). As a second-line treatment, the PD-1 inhibitor pembrolizumab has not only targeted PD-1 highly expressed on the surface of HIV-related tumors but has also shown potential in promoting immune reconstitution in HIV-associated tumors [88]. According to a retrospective study by the National Cancer Institute, among four HIV-associated DLBCL patients treated solely with the PD-1 inhibitor pembrolizumab, two out of four achieved partial remission (PR) after six cycles of treatment, one out of four had stable disease (SD) after seven cycles of treatment, and one out of four died due to bone marrow infiltration after three cycles of treatment. This study preliminarily demonstrates the safety of pembrolizumab in relapsed and refractory HIV-related DLBCL patients, indicating that even in patients with relatively low CD4^+^ T-cell counts (CD4^+^ T cells < 50 cells/μL), partial remission can be observed.

CTLA-4 inhibitors, with ipilimumab as a representative medication, have been studied in clinical trials such as NCT02408861, which investigates the safety and efficacy of ipilimumab in combination with pomalidomide in HIV-associated classical HL [86].

Immune checkpoint inhibitors have shown efficacy with fewer adverse events, but a limitation is their low response rate as monotherapy. Researchers are actively studying the mechanisms of immune checkpoint inhibitor resistance and exploring combination treatment regimens to enhance their effectiveness. Studies have suggested that immune checkpoint inhibitors may have reduced efficacy in DLBCL with MYC overexpression or loss of HLA [87,88]. Therefore, testing the mutational profiles of HAL may help predict the effectiveness of immune checkpoint blockade therapy [87].

### 4.3. Adoptive Cellular Immunotherapies

Adoptive immunotherapies, including hematopoietic stem cell transplantation (HSCT) and chimeric antigen receptor T-cell therapy (CAR-T), have shown promise in the treatment of lymphoma. Allo-HSCT involves the infusion of stem cells from a donor to restore the patient’s immune system. In the case of HAL, specific consideration is given to selecting donors with a CCR5Δ32 mutation, as this mutation confers resistance to HIV infection. So far, stem cell transplantation with the CCR5Δ32 gene deletion has successfully cured five cases of HIV-associated hematological cancer. In addition, recent research has reported a case of an HIV-infected individual successfully cured for the first time with stem cell transplantation without the CCR5 deletion, indicating that the CCR5Δ32 gene deletion in stem cell transplantation is not a necessary condition for achieving long-term HIV remission or cure. This will make it easier for more HIV-infected individuals who require stem cell transplantation to treat cancer to find suitable donors, thus alleviating their condition and extending their lives. While allo-HSCT can be curative, its widespread application is challenging due to the complexities of transplantation and the lack of suitable donors [89].

CAR-T therapy is another form of adoptive immunotherapy that has shown promising results in lymphoma treatment. Currently, CAR-T therapies targeting CD19 have been approved globally. The efficacy of CAR-T therapies in treating HAL has also been explored and summarized in Table 2. For HAL, current data are primarily focused on case reports because PLWH have not been included in pivotal trials. As of now, there have been a total of six case reports of HAL patients who underwent CAR-T therapy. Among them, three patients achieved complete remission (CR), and one patient achieved partial remission (PR) [90]. Despite four patients experiencing cytokine release syndrome (CRS) or grade 3–4 immune effector cell-associated neurotoxicity syndrome (ICAN), the severity of symptoms was relatively mild. This outcome suggests that CAR-T cell therapy may have certain efficacy in HAL patients, although vigilance toward immune-related adverse reactions is still warranted during the treatment process. With the progress of research, more data will contribute to the evaluation of the long-term effectiveness and safety of CAR-T therapy in treating lymphomas associated with HIV infection.

**Table 2:** Immune checkpoint inhibitors in the treatment of HIV-associated lymphoma.

Drug	Research Type	Lymphoma Subtype	Treatment Options	Outcomes	Reference
Nivolumab (Nivo)	Case report	Relapsed/refractory classical Hodgkin lymphoma	Nivo 3 mg/kg q 2 wk	Partial remission was achieved after 5 months (10 doses) of Navulizumab treatment	[91]
Nivolumab (Nivo)	Prospective cohort study	Relapsed/refractory classical Hodgkin lymphoma	Nivo 3 mg/kg q 2 wk	Incidence of infectious complications was 10% with the median time of onset—98 days; OS at 1 year after first Nivo administration was 96.5%	[92]
Nivolumab Ipilimumab (Ipi)	A phase I study (NCT02408861)	Classical Hodgkin lymphoma	Nivo 3 mg/kg q 2 wk. Nivo 240 mg q 2 wk + Ipi 1 mg/kg q 6 wk	NA	[86]
Pembrolizumab (Pemb)	Retrospective study	Diffuse large B-cell lymphoma; primary effusion lymphoma; plasmablastic lymphoma	Pemb 200 mg q 3 wk. Pemb 200 mg q 4 wk + pomalidomide 4 mg q.d.	PFS was 4.1 months; OS was 14.7 months; there were four irAEs, all CTCAEv5 grade 2–3; no irAEs occurred in patients receiving the combination of pemb and pomalidomide.	[93]
Pembrolizumab (Pemb)	A phase I study (NCT02595866)	Non- Hodgkin lymphoma	Pemb 200 mg q 3 wk	Partial response in 2 participants with NHL, 1 participant with DLBCL, 1 participant with primary effusion lymphoma	[94]

OS = overall survival; NA = not applicable; irAEs = immune-related adverse events; q = every; wk = week; d = days.

## 5. Discussion

Abundant basic research indicates that the occurrence and development of HIV-associated lymphoma are indirectly related to CD4^+^ T-cell damage after HIV infection, chronic B-cell activation, and oncovirus coinfection, as well as directly related to proteins and RNA encoded by HIV. Currently, diverse treatment options are being explored for HAL, including monoclonal antibodies, immune checkpoint inhibitors, and adoptive immunotherapy. These approaches aim to address the specific challenges posed by the immunosuppressive state of PLWH and provide tailored treatments for HAL. In clinical practice, it has been found that immunotherapy is safe and feasible for HIV-associated lymphoma and holds great potential. However, these clinical studies often have small sample sizes. Therefore, further research, particularly large-scale clinical trials and investigations into disease pathogenesis and heterogeneity, is necessary to advance our understanding of HAL and improve treatment outcomes. The exploration of various therapeutic strategies and the knowledge gained from studying HAL can also have implications for the broader field of lymphoma research.

## Figures and Tables

**Table 1:** The first-line therapy for HIV-associated lymphoma.

Subtypes of HIV- Associated Lymphomas	Preferred Regimens	Other Recommended Regimens
Diffuse large B-cell lymphoma	R-EPOCH	RCHOP
Primary effusion lymphoma	R-EPOCH	RCHOP
Burkitt lymphoma	CODOX-M/IVAC (modified) or DA-EPOCH-R	R-Hyper CVAD
Plasmablastic lymphoma	EPOCH (preferred)	CODOX-M/IVAC (modified) or R-HyperCVAD

R-EPOCH: rituximab plus etoposide, prednisone, vincristine, cyclophosphamide, and doxorubicin; R-CHOP: rituximab, cyclophosphamide, doxorubicin, vincristine, and prednisone; CODOX-M/IVAC (modified): modification of cyclophosphamide, vincristine, doxorubicin, high-dose methotrexate/ifosfamide, etoposide, and high-dose cytarabine; DA-R-EPOCH: dose-adjusted rituximab plus etoposide, prednisone, vincristine, cyclophosphamide, and doxorubicin; R-Hyper CVAD: rituximab plus hyper fractionated cyclophosphamide, vincristine, doxorubicin, and dexamethasone.

## Data Availability

Not applicable.

## References

[1] Carbone A., Vaccher E., Gloghini A. (2022). Hematologic cancers in individuals infected by HIV. Blood.

[2] Alaggio R., Amador C., Anagnostopoulos I., Attygalle A.D., Araujo I.B.O., Berti E., Bhagat G., Borges A.M., Boyer D., Calaminici M. (2022). The 5th edition of the World Health Organization Classification of Haematolymphoid Tumours: Lymphoid Neoplasms. Leukemia.

[3] Isaguliants M., Bayurova E., Avdoshina D., Kondrashova A., Chiodi F., Palefsky J.M. (2021). Oncogenic Effects of HIV-1 Proteins, Mechanisms Behind. Cancers.

[4] Feng Y., Wang Z., Zeng D., Song S., Yang Y., Wang A., Xu J., Guo W., Wu M., Shi Y. (2022). High expression of HIV-1 matrix protein p17 in both lymphoma and lymph node tissues of AIDS patients. Pathol. Res. Pract..

[5] Carroll V.A., Lafferty M.K., Marchionni L., Bryant J.L., Gallo R.C., Garzino-Demo A. (2016). Expression of HIV-1 matrix protein p17 and association with B-cell lymphoma in HIV-1 transgenic mice. Proc. Natl. Acad. Sci. USA.

[6] Bugatti A., Caccuri F., Filippini F., Ravelli C., Caruso A. (2021). Binding to PI(4,5)P(2) is indispensable for secretion of B-cell clonogenic HIV-1 matrix protein p17 variants. J. Biol. Chem..

[7] Giagulli C., Caccuri F., Zorzan S., Bugatti A., Zani A., Filippini F., Manocha E., D’Ursi P., Orro A., Dolcetti R. (2021). B-cell clonogenic activity of HIV-1 p17 variants is driven by PAR1-mediated EGF transactivation. Cancer Gene Ther..

[8] Alves de Souza Rios L., Mapekula L., Mdletshe N., Chetty D., Mowla S. (2021). HIV-1 Transactivator of Transcription (Tat) Co-operates with AP-1 Factors to Enhance c-MYC Transcription. Front. Cell Dev. Biol..

[9] Valyaeva A.A., Tikhomirova M.A., Potashnikova D.M., Bogomazova A.N., Snigiryova G.P., Penin A.A., Logacheva M.D., Arifulin E.A., Shmakova A.A., Germini D. (2022). Ectopic expression of HIV-1 Tat modifies gene expression in cultured B cells: Implications for the development of B-cell lymphomas in HIV-1-infected patients. PeerJ.

[10] Sall F.B., El Amine R., Markozashvili D., Tsfasman T., Oksenhendler E., Lipinski M., Vassetzky Y., Germini D. (2019). HIV-1 Tat protein induces aberrant activation of AICDA in human B-lymphocytes from peripheral blood. J. Cell. Physiol..

[11] Akbay B., Germini D., Bissenbaev A.K., Musinova Y.R., Sheval E.V., Vassetzky Y., Dokudovskaya S. (2021). HIV-1 Tat Activates Akt/mTORC1 Pathway and AICDA Expression by Downregulating Its Transcriptional Inhibitors in B Cells. Int. J. Mol. Sci..

[12] Germini D., Tsfasman T., Klibi M., El-Amine R., Pichugin A., Iarovaia O.V., Bilhou-Nabera C., Subra F., Bou Saada Y., Sukhanova A. (2017). HIV Tat induces a prolonged MYC relocalization next to IGH in circulating B-cells. Leukemia.

[13] Mdletshe N., Nel A., Shires K., Mowla S. (2020). HIV Nef enhances the expression of oncogenic c-MYC and activation-induced cytidine deaminase in Burkitt lymphoma cells, promoting genomic instability. Infect. Agents Cancer.

[14] De Mel S., Hue S.S., Jeyasekharan A.D., Chng W.J., Ng S.B. (2019). Molecular pathogenic pathways in extranodal NK/T cell lymphoma. J. Hematol. Oncol..

[15] Park J.E., Kim T.S., Zeng Y., Mikolaj M., Il Ahn J., Alam M.S., Monnie C.M., Shi V., Zhou M., Chun T.W. (2024). Centrosome amplification and aneuploidy driven by the HIV-1-induced Vpr•VprBP•Plk4 complex in CD4^+^ T cells. Nat. Commun..

[16] Mellors J.W., Guo S., Naqvi A., Brandt L.D., Su L., Sun Z., Joseph K.W., Demirov D., Halvas E.K., Butcher D. (2021). Insertional activation of STAT3 and LCK by HIV-1 proviruses in T cell lymphomas. Sci. Adv..

[17] Armani-Tourret M., Bone B., Tan T.S., Sun W., Bellefroid M., Struyve T., Louella M., Yu X.G., Lichterfeld M. (2024). Immune targeting of HIV-1 reservoir cells: A path to elimination strategies and cure. Nat. Rev. Microbiol..

[18] Veenhuis R.T., Abreu C.M., Shirk E.N., Gama L., Clements J.E. (2021). HIV replication and latency in monocytes and macrophages. Semin. Immunol..

[19] Sáez-Cirión A., Sereti I. (2021). Immunometabolism and HIV-1 pathogenesis: Food for thought. Nat. Rev. Immunol..

[20] Singh H., Jadhav S., Arif Khan A., Aggarwal S.K., Choudhari R., Verma S., Aggarwal S., Gupta V., Singh A., Nain S. (2022). APOBEC3, TRIM5α, and BST2 polymorphisms in healthy individuals of various populations with special references to its impact on HIV transmission. Microb. Pathog..

[21] Singh H., Samani D., Ghate M.V., Gangakhedkar R.R. (2018). Impact of cellular restriction gene (TRIM5α, BST-2) polymorphisms on the acquisition of HIV-1 and disease progression. J. Gene Med..

[22] Mitchell R.S., Katsura C., Skasko M.A., Fitzpatrick K., Lau D., Ruiz A., Stephens E.B., Margottin-Goguet F., Benarous R., Guatelli J.C. (2009). Vpu antagonizes BST-2-mediated restriction of HIV-1 release via beta-TrCP and endo-lysosomal trafficking. PLoS Pathog..

[23] Lê-Bury G., Niedergang F. (2018). Defective Phagocytic Properties of HIV-Infected Macrophages: How Might They Be Implicated in the Development of Invasive Salmonella Typhimurium?. Front. Immunol..

[24] Wang L., Li G., Yao Z.Q., Moorman J.P., Ning S. (2015). MicroRNA regulation of viral immunity, latency, and carcinogenesis of selected tumor viruses and HIV. Rev. Med. Virol..

[25] Adu-Gyamfi C.G., Savulescu D., George J.A., Suchard M.S. (2019). Indoleamine 2, 3-Dioxygenase-Mediated Tryptophan Catabolism: A Leading Star or Supporting Act in the Tuberculosis and HIV Pas-de-Deux?. Front. Cell. Infect. Microbiol..

[26] Planès R., Bahraoui E. (2013). HIV-1 Tat protein induces the production of IDO in human monocyte derived-dendritic cells through a direct mechanism: Effect on T cells proliferation. PLoS ONE.

[27] Favre D., Mold J., Hunt P.W., Kanwar B., Loke P., Seu L., Barbour J.D., Lowe M.M., Jayawardene A., Aweeka F. (2010). Tryptophan catabolism by indoleamine 2,3-dioxygenase 1 alters the balance of TH17 to regulatory T cells in HIV disease. Sci. Transl. Med..

[28] Terness P., Bauer T.M., Röse L., Dufter C., Watzlik A., Simon H., Opelz G. (2002). Inhibition of allogeneic T cell proliferation by indoleamine 2,3-dioxygenase-expressing dendritic cells: Mediation of suppression by tryptophan metabolites. J. Exp. Med..

[29] Judge C.J., Kostadinova L., Sherman K.E., Butt A.A., Falck-Ytter Y., Funderburg N.T., Landay A.L., Lederman M.M., Sieg S.F., Sandberg J.K. (2017). CD56(bright) NK IL-7Rα expression negatively associates with HCV level, and IL-7-induced NK function is impaired during HCV and HIV infections. J. Leukoc. Biol..

[30] Lucar O., Reeves R.K., Jost S. (2019). A Natural Impact: NK Cells at the Intersection of Cancer and HIV Disease. Front. Immunol..

[31] Zhang Z., Zhou Y., Lu J., Chen Y.F., Hu H.Y., Xu X.Q., Fu G.F. (2021). Changes in NK Cell Subsets and Receptor Expressions in HIV-1 Infected Chronic Patients and HIV Controllers. Front. Immunol..

[32] Fromentin R., Bakeman W., Lawani M.B., Khoury G., Hartogensis W., DaFonseca S., Killian M., Epling L., Hoh R., Sinclair E. (2016). CD4^+^ T Cells Expressing PD-1, TIGIT and LAG-3 Contribute to HIV Persistence during ART. PLoS Pathog..

[33] Mirzaei R., Gordon A., Zemp F.J., Kumar M., Sarkar S., Luchman H.A., Bellail A.C., Hao C., Mahoney D.J., Dunn J.F. (2021). PD-1 independent of PD-L1 ligation promotes glioblastoma growth through the NFκB pathway. Sci. Adv..

[34] Demers K.R., Makedonas G., Buggert M., Eller M.A., Ratcliffe S.J., Goonetilleke N., Li C.K., Eller L.A., Rono K., Maganga L. (2016). Temporal Dynamics of CD8+ T Cell Effector Responses during Primary HIV Infection. PLoS Pathog..

[35] Pan Y., Zhang Z.N., Yin L.B., Fu Y.J., Jiang Y.J., Shang H. (2019). Reduced eIF3d accelerates HIV disease progression by attenuating CD8^+^ T cell function. J. Transl. Med..

[36] Shmakova A., Hugot C., Kozhevnikova Y., Schwager Karpukhina A., Tsimailo I., Gérard L., Boutboul D., Oksenhendler E., Szewczyk-Roszczenko O., Roszczenko P. (2024). Chronic HIV-1 Tat action induces HLA-DR downregulation in B cells: A mechanism for lymphoma immune escape in people living with HIV. J. Med. Virol..

[37] Doyon-Laliberté K., Aranguren M., Poudrier J., Roger M. (2022). Marginal Zone B-Cell Populations and Their Regulatory Potential in the Context of HIV and Other Chronic Inflammatory Conditions. Int. J. Mol. Sci..

[38] Doyon-Laliberté K., Aranguren M., Byrns M., Chagnon-Choquet J., Paniconi M., Routy J.P., Tremblay C., Quintal M.C., Brassard N., Kaufmann D.E. (2022). Excess BAFF Alters NR4As Expression Levels and Breg Function of Human Precursor-like Marginal Zone B-Cells in the Context of HIV-1 Infection. Int. J. Mol. Sci..

[39] Münz C. (2019). Latency and lytic replication in Epstein-Barr virus-associated oncogenesis. Nat. Rev. Microbiol..

[40] Lurain K., Ramaswami R., Yarchoan R. (2022). The role of viruses in HIV-associated lymphomas. Semin. Hematol..

[41] McHugh D., Myburgh R., Caduff N., Spohn M., Kok Y.L., Keller C.W., Murer A., Chatterjee B., Rühl J., Engelmann C. (2020). EBV renders B cells susceptible to HIV-1 in humanized mice. Life Sci. Alliance.

[42] Katsuya H., Cook L.B.M., Rowan A.G., Melamed A., Turpin J., Ito J., Islam S., Miyazato P., Jek Yang Tan B., Matsuo M. (2022). Clonality of HIV-1- and HTLV-1-Infected Cells in Naturally Coinfected Individuals. J. Infect. Dis..

[43] Binnewies M., Roberts E.W., Kersten K., Chan V., Fearon D.F., Merad M., Coussens L.M., Gabrilovich D.I., Ostrand-Rosenberg S., Hedrick C.C. (2018). Understanding the tumor immune microenvironment (TIME) for effective therapy. Nat. Med..

[44] Liu Y., Zhou X., Wang X. (2021). Targeting the tumor microenvironment in B-cell lymphoma: Challenges and opportunities. J. Hematol. Oncol..

[45] Taylor J.G., Liapis K., Gribben J.G. (2015). The role of the tumor microenvironment in HIV-associated lymphomas. Biomark. Med..

[46] Hernández-Ramírez R.U., Qin L., Lin H., Leyden W., Neugebauer R.S., Althoff K.N., Achenbach C.J., Hessol N.A., D’Souza G., Gebo K.A. (2019). Association of immunosuppression and HIV viraemia with non-Hodgkin lymphoma risk overall and by subtype in people living with HIV in Canada and the USA: A multicentre cohort study. Lancet HIV.

[47] Gopal S., Patel M.R., Yanik E.L., Cole S.R., Achenbach C.J., Napravnik S., Burkholder G.A., Reid E.G., Rodriguez B., Deeks S.G. (2013). Temporal trends in presentation and survival for HIV-associated lymphoma in the antiretroviral therapy era. J. Natl. Cancer Inst..

[48] Chapman J.R., Bouska A.C., Zhang W., Alderuccio J.P., Lossos I.S., Rimsza L.M., Maguire A., Yi S., Chan W.C., Vega F. (2021). EBV-positive HIV-associated diffuse large B cell lymphomas are characterized by JAK/STAT (STAT3) pathway mutations and unique clinicopathologic features. Br. J. Haematol..

[49] Nowakowski G.S., Feldman T., Rimsza L.M., Westin J.R., Witzig T.E., Zinzani P.L. (2019). Integrating precision medicine through evaluation of cell of origin in treatment planning for diffuse large B-cell lymphoma. Blood Cancer J..

[50] Besson C., Lancar R., Prevot S., Algarte-Genin M., Delobel P., Bonnet F., Meyohas M.C., Partisani M., Oberic L., Gabarre J. (2017). Outcomes for HIV-associated diffuse large B-cell lymphoma in the modern combined antiretroviral therapy era. Aids.

[51] Baptista M.J., Tapia G., Morgades M., Muncunill J., Muñoz-Marmol A.M., Montoto S., Gribben J.G., Calaminici M., Martinez A., Gonzalez-Farre B. (2019). Using the Lymph2Cx assay for assessing cell-of-origin subtypes of HIV-related diffuse large B-cell lymphoma. Leuk. Lymphoma.

[52] Capello D., Scandurra M., Poretti G., Rancoita P.M., Mian M., Gloghini A., Deambrogi C., Martini M., Rossi D., Greiner T.C. (2010). Genome wide DNA-profiling of HIV-related B-cell lymphomas. Br. J. Haematol..

[53] Chen J., Sun L., Dai Y., Zhang L., Yang K., Han X., Ding X., Gao H., Zhou X., Wang P. (2023). Clinical pathology of primary central nervous system lymphoma in HIV-positive patients—A 41 Chinese patients retrospective study. Ann. Diagn. Pathol..

[54] Chao C., Silverberg M.J., Martínez-Maza O., Chi M., Abrams D.I., Haque R., Zha H.D., McGuire M., Xu L., Said J. (2012). Epstein-Barr virus infection and expression of B-cell oncogenic markers in HIV-related diffuse large B-cell Lymphoma. Clin. Cancer Res..

[55] Liapis K., Clear A., Owen A., Coutinho R., Greaves P., Lee A.M., Montoto S., Calaminici M., Gribben J.G. (2013). The microenvironment of AIDS-related diffuse large B-cell lymphoma provides insight into the pathophysiology and indicates possible therapeutic strategies. Blood.

[56] Baptista M.J., Tapia G., Muñoz-Marmol A.M., Muncunill J., Garcia O., Montoto S., Gribben J.G., Calaminici M., Martinez A., Veloza L. (2022). Genetic and phenotypic characterisation of HIV-associated aggressive B-cell non-Hodgkin lymphomas, which do not occur specifically in this population: Diagnostic and prognostic implications. Histopathology.

[57] Maguire A., Chen X., Wisner L., Malasi S., Ramsower C., Kendrick S., Barrett M.T., Glinsmann-Gibson B., McGrath M., Rimsza L.M. (2019). Enhanced DNA repair and genomic stability identify a novel HIV-related diffuse large B-cell lymphoma signature. Int. J. Cancer.

[58] Guech-Ongey M., Simard E.P., Anderson W.F., Engels E.A., Bhatia K., Devesa S.S., Mbulaiteye S.M. (2010). AIDS-related Burkitt lymphoma in the United States: What do age and CD4 lymphocyte patterns tell us about etiology and/or biology?. Blood.

[59] Sapkota S., Shaikh H. (2024). Non-Hodgkin Lymphoma. StatPearls.

[60] Han X., Jemal A., Hulland E., Simard E.P., Nastoupil L., Ward E., Flowers C.R. (2017). HIV Infection and Survival of Lymphoma Patients in the Era of Highly Active Antiretroviral Therapy. Cancer Epidemiol. Biomark. Prev..

[61] Mbulaiteye S.M., Pullarkat S.T., Nathwani B.N., Weiss L.M., Rao N., Emmanuel B., Lynch C.F., Hernandez B., Neppalli V., Hawes D. (2014). Epstein-Barr virus patterns in US Burkitt lymphoma tumors from the SEER residual tissue repository during 1979–2009. Apmis.

[62] Cesarman E. (2013). Pathology of lymphoma in HIV. Curr. Opin. Oncol..

[63] Ajasin D., Eugenin E.A. (2020). HIV-1 Tat: Role in Bystander Toxicity. Front. Cell. Infect. Microbiol..

[64] Shmakova A., Tsimailo I., Kozhevnikova Y., Gérard L., Boutboul D., Oksenhendler E., Tuaillon E., Rivault A., Germini D., Vassetzky Y. (2024). HIV-1 Tat is present in the serum of people living with HIV-1 despite viral suppression. Int. J. Infect. Dis..

[65] Ramorola B.R., Goolam-Hoosen T., Alves de Souza Rios L., Mowla S. (2021). Modulation of Cellular MicroRNA by HIV-1 in Burkitt Lymphoma Cells—A Pathway to Promoting Oncogenesis. Genes.

[66] Zelenetz A.D., Gordon L.I., Abramson J.S., Advani R.H., Andreadis B., Bartlett N.L., Budde L.E., Caimi P.F., Chang J.E., Christian B. (2023). NCCN Guidelines^®^ Insights: B-Cell Lymphomas, Version 6. J. Natl. Compr. Cancer Netw..

[67] Biggar R.J., Horm J., Goedert J.J., Melbye M. (1987). Cancer in a group at risk of acquired immunodeficiency syndrome (AIDS) through 1984. Am. J. Epidemiol..

[68] Carroll V., Garzino-Demo A. (2015). HIV-associated lymphoma in the era of combination antiretroviral therapy: Shifting the immunological landscape. Pathog. Dis..

[69] Hleyhel M., Hleyhel M., Bouvier A.M., Belot A., Tattevin P., Pacanowski J., Genet P., De Castro N., Berger J.L., Dupont C. (2014). Risk of non-AIDS-defining cancers among HIV-1-infected individuals in France between 1997 and 2009: Results from a French cohort. Aids.

[70] Kowalkowski M.A., Mims M.A., Day R.S., Du X.L., Chan W., Chiao E.Y. (2014). Longer duration of combination antiretroviral therapy reduces the risk of Hodgkin lymphoma: A cohort study of HIV-infected male veterans. Cancer Epidemiol..

[71] Said J.W. (2007). Immunodeficiency-related Hodgkin lymphoma and its mimics. Adv. Anat. Pathol..

[72] Carbone A., Gloghini A., Serraino D., Spina M. (2009). HIV-associated Hodgkin lymphoma. Curr. Opin. HIV AIDS.

[73] Koulis A., Trivedi P., Ibrahim H., Bower M., Naresh K.N. (2014). The role of the microenvironment in human immunodeficiency virus-associated classical Hodgkin lymphoma. Histopathology.

[74] Hartmann S., Jakobus C., Rengstl B., Döring C., Newrzela S., Brodt H.R., Wolf T., Hansmann M.L. (2013). Spindle-shaped CD163+ rosetting macrophages replace CD4^+^ T-cells in HIV-related classical Hodgkin lymphoma. Mod. Pathol..

[75] Grywalska E., Markowicz J., Grabarczyk P., Pasiarski M., Roliński J. (2013). Epstein-Barr virus-associated lymphoproliferative disorders. Postep. Hig. Med. Dosw..

[76] Carbone A., Gloghini A., Caruso A., De Paoli P., Dolcetti R. (2017). The impact of EBV and HIV infection on the microenvironmental niche underlying Hodgkin lymphoma pathogenesis. Int. J. Cancer.

[77] De Paoli P., Carbone A. (2015). Microenvironmental abnormalities induced by viral cooperation: Impact on lymphomagenesis. Semin. Cancer Biol..

[78] Caccuri F., Giagulli C., Bugatti A., Benetti A., Alessandri G., Ribatti D., Marsico S., Apostoli P., Slevin M.A., Rusnati M. (2012). HIV-1 matrix protein p17 promotes angiogenesis via chemokine receptors CXCR1 and CXCR2. Proc. Natl. Acad. Sci. USA.

[79] Eliopoulos A.G., Stack M., Dawson C.W., Kaye K.M., Hodgkin L., Sihota S., Rowe M., Young L.S. (1997). Epstein-Barr virus-encoded LMP1 and CD40 mediate IL-6 production in epithelial cells via an NF-kappaB pathway involving TNF receptor-associated factors. Oncogene.

[80] Re A., Cattaneo C., Rossi G. (2019). Hiv and Lymphoma: From Epidemiology to Clinical Management. Mediterr. J. Hematol. Infect. Dis..

[81] Rezahosseini O., Hanaei S., Hamadani M., Keshavarz-Fathi M., Rezaei N. (2018). The promising role of monoclonal antibodies for immunotherapy of the HIV-associated cancer, non-Hodgkin lymphoma. Int. Rev. Immunol..

[82] Im A., Pavletic S.Z. (2017). Immunotherapy in hematologic malignancies: Past, present, and future. J. Hematol. Oncol..

[83] Schommers P., Gillor D., Hentrich M., Wyen C., Wolf T., Oette M., Zoufaly A., Wasmuth J.C., Bogner J.R., Müller M. (2018). Incidence and risk factors for relapses in HIV-associated non-Hodgkin lymphoma as observed in the German HIV-related lymphoma cohort study. Haematologica.

[84] Ramaswami R., Lurain K., Peer C.J., Serquiña A., Wang V., Widell A., Goncalves P., Steinberg S.M., Marshall V., George J. (2020). Tocilizumab in patients with symptomatic Kaposi sarcoma herpesvirus-associated multicentric Castleman disease. Blood.

[85] Cozzi I., Rossi G., Rullo E., Ascoli V. (2022). Classic KSHV/HHV-8-positive Primary Effusion Lymphoma (PEL): A Systematic Review and Meta-Analysis of Case Reports. Mediterr. J. Hematol. Infect. Dis..

[86] Rajdev L., Chiao E.Y., Lensing S., Little R.F., Dittmer D., Einstein M.H., Haigentz M., Sparano J.A., Mitsuyasu R.T. (2018). AMC 095 (AIDS Malignancy Consortium): A phase I study of ipilimumab (IPI) and nivolumab (NIVO) in advanced HIV associated solid tumors (ST) with expansion cohorts in HIV associated solid tumors and classical Hodgkin lymphoma (cHL). J. Clin. Oncol..

[87] Nijland M., Veenstra R.N., Visser L., Xu C., Kushekhar K., van Imhoff G.W., Kluin P.M., van den Berg A., Diepstra A. (2017). HLA dependent immune escape mechanisms in B-cell lymphomas: Implications for immune checkpoint inhibitor therapy?. Oncoimmunology.

[88] Landsburg D.J., Koike A., Nasta S.D., Svoboda J., Schuster S.J., Wasik M.A., Caponetti G.C. (2021). Patterns of immune checkpoint protein expression in MYC-overexpressing aggressive B-cell non-Hodgkin lymphomas. Cancer Immunol. Immunother..

[89] Chen X., Jia L., Zhang X., Zhang T., Zhang Y. (2022). One arrow for two targets: Potential co-treatment regimens for lymphoma and HIV. Blood Rev..

[90] Hattenhauer S.T., Mispelbaum R., Hentrich M., Boesecke C., Monin M.B. (2023). Enabling CAR T-cell therapies for HIV-positive lymphoma patients—A call for action. HIV Med..

[91] Sandoval-Sus J.D., Mogollon-Duffo F., Patel A., Visweshwar N., Laber D.A., Kim R., Jagal M.V. (2017). Nivolumab as salvage treatment in a patient with HIV-related relapsed/refractory Hodgkin lymphoma and liver failure with encephalopathy. J. Immunother. Cancer.

[92] Rogacheva Y., Popova M., Lepik K., Kondakova E., Zalyalov Y., Stelmah L., Volkova A., Nikolaev I., Goloshchapov O., Barhatov I. (2019). INFECTIOUS COMPLICATIONS OF NIVOLUMAB THERAPY IN RELAPSED/REFRACTORY HODGKIN’S LYMPHOMA. Hematol. Oncol..

[93] Lurain K., Ramaswami R., Mangusan R., Widell A., Ekwede I., George J., Ambinder R., Cheever M., Gulley J.L., Goncalves P.H. (2021). Use of pembrolizumab with or without pomalidomide in HIV-associated non-Hodgkin’s lymphoma. J. Immunother. Cancer.

[94] Uldrick T.S., Gonçalves P.H., Abdul-Hay M., Claeys A.J., Emu B., Ernstoff M.S., Fling S.P., Fong L., Kaiser J.C., Lacroix A.M. (2019). Assessment of the Safety of Pembrolizumab in Patients with HIV and Advanced Cancer-A Phase 1 Study. JAMA Oncol..

